# Correlates of Nine-Month Retention following Interim Buprenorphine-Naloxone Treatment in Opioid Dependence: A Pilot Study

**DOI:** 10.1155/2016/6487217

**Published:** 2016-01-21

**Authors:** A. Håkansson, C. Widinghoff, T. Abrahamsson, C. Gedeon

**Affiliations:** ^1^Department of Clinical Sciences Lund, Division of Psychiatry, Lund University, 221 85 Lund, Sweden; ^2^Malmö Addiction Center, Department of Psychiatry, 205 02 Malmö, Skane Region, Sweden; ^3^Solstenen Outpatient Unit for Opiate Maintenance Treatment, Östra Mårtensgatan 15, 223 61 Lund, Sweden

## Abstract

Interim medication-only treatment has been suggested for the initiation of opioid maintenance treatment (OMT) in opioid-dependent subjects, but this rarely has been studied using buprenorphine instead of methadone. Following a pilot trial assessing interim buprenorphine-naloxone treatment in order to facilitate transfer into OMT, we here aimed to study retention, and potential correlates of retention, in full-scale treatment. Thirty-six patients successfully referred from a waiting list through an interim treatment phase were followed for nine months in OMT. Baseline characteristics, as well as urine analyses during the interim phase and during full-scale OMT, were studied as potential correlates of retention. The nine-month retention in OMT was 83 percent (*n* = 30). While interim-phase urine samples positive for benzodiazepines did not significantly predict dropout from full-scale OMT (*p* = 0.09), urine samples positive for benzodiazepines within full-scale OMT were significantly associated with dropout (*p* < 0.01), in contrast to other substances and baseline characteristics. Retention remained high through nine months in this pilot study sample of patients referred through buprenorphine-naloxone interim treatment, but use of benzodiazepines is problematic, and the present data suggest that it may be associated with treatment dropout.

## 1. Introduction

Evidence-based treatment of opioid dependence is based on opioid maintenance treatment (OMT) with methadone, buprenorphine, or buprenorphine-naloxone [1], the latter aiming to decrease the abuse potential of the medication [2].

A few previous trials have assessed a temporary, medication-only strategy, an interim medication model, aiming to facilitate treatment entry and to improve patients' outcome compared to waiting list or other nontreatment conditions. While these interventions with an interim methadone medication generally have been satisfactory [3–7], a potentially safer interim treatment strategy, with respect to overdose risk [8], would be to use buprenorphine during the medication-only phase, but this rarely has been tested. An exception is a Norwegian study demonstrating a 29 percent retention over three months with interim buprenorphine [9], compared to only 2 percent in the control condition.

In the pilot study preceding the present follow-up study, it was demonstrated that 57 percent of opioid-dependent patients on a waiting list were successfully referred to full-scale OMT through an interim, medication-only phase with buprenorphine-naloxone [10]. Patients were successfully transferred if they were able to become drug-free after provision of an available treatment slot, and they were considered to be dropouts if they failed to become drug-free at that time, or if they were discharged due to failure to show up for treatment or due to an intense polydrug use pattern constituting a medical risk to the patient. While that study demonstrated a relative effectiveness of the interim model using buprenorphine-naloxone in the interim phase, it was also shown that failure to enter the full-scale treatment condition was associated with a higher score on the Alcohol Use Disorders Identification Test (AUDIT) [11].

Polydrug use is highly prevalent in heroin-dependent patients, and it has been described that the use of other drugs, in addition to opiates, increases the risk of overdoses [12, 13]. Continued use of other drugs is very common in patients in OMT [14]; in a German study it was reported that 90 percent of treated patients used at least one drug in addition to opiates [15]. The polydrug use pattern commonly involves benzodiazepines, the use of which has been reported in 46–71 percent of treated subjects [16, 17], and cannabis, often used by a majority of treated patients [18–23]. Likewise, many opiate-dependent patients in treatment use cocaine and other stimulants [18–20, 22, 24–26]. The possibly deteriorating role of continued polydrug use in the course of treatment for opiate dependence has been discussed in previous literature, with ambiguous results, indicating that continued polydrug use may increase the risk of continued heroin use [16, 17, 24, 25, 27], whereas treatment retention in different studies has been reported to be either lowered [14, 27–29] or unaffected by polydrug use [16, 17, 21, 23, 27].

Retention in OMT for patients referred through a buprenorphine-naloxone interim condition has not been evaluated, and there is need to explore, in a pilot study, its effectiveness in retaining patients after actual entry to full-scale OMT, and potential factors associated with retention. Thus, the present follow-up study of the initial pilot study aimed (1) to assess nine-month retention in OMT after being transferred from interim treatment with buprenorphine-naloxone, and (2) to identify potential baseline and treatment correlates of dropout during subsequent full-scale treatment, including how urine drug tests during the interim phase and during the full-scale OMT phase were associated with dropout.

## 2. Method 

The sample consisted of participants from the pilot study “Interim Buprenorphine Study Lund.” The initial pilot study included 44 patients enrolled up to the termination of the pilot project [10], among whom 25 individuals were successfully transferred from interim treatment to full-scale OMT. At the time of follow-up, the overall project had included a total of 57 individuals, among whom 40 (70%) had successfully entered the full-scale treatment. In total, 36 patients had entered the full-scale treatment through the interim condition and had at least nine months of possible follow-up time and constitute the cohort assessed in the present retention study. Based on the inclusion criteria of the pilot study and the continued clinical project, all patients were opiate-dependent according to the ICD-10 and met the criteria for OMT as stated by the Swedish regulations (opioid dependence with a documented use of illicit opiates for at least one year, and absence of use of other drugs comprising a medical risk). Clients were not eligible for the study if they had a medical contraindication to buprenorphine, if treatment with methadone was considered necessary by the treating physician (e.g., due to primary methadone dependence), or if they suffered from an unstable psychiatric disease, such as acute psychotic disorders or active suicidal behavior. The study was carried out in a specialized OMT facility in Lund, Sweden. According to national Swedish regulations, methadone or buprenorphine maintenance treatment is available only in specialized facilities where supervised dosing is provided during the first phase of the treatment, with gradually increasing possibilities of take-away doses after six months. OMT facilities in Sweden typically provide counselling as needed, along with methadone and buprenorphine treatment, and typically collaborate with social authorities for patients in need of that, such as patients in unstable housing situations.

The study was approved by the regional ethics committee of Lund, Sweden (file number 2010/596), and all participants signed an informed consent.

Data were derived from the Addiction Severity Index [30] administered by the social services prior to study start, and substance use was reported in additional questionnaires administered at the OMT facility prior to study start: the AUDIT [11] and a short survey with additional questions about substance-specific drug use.

As described previously [10], the interim phase, an alternative to a waiting list situation, was a medication-only treatment strategy aiming to stabilize included patients in order to facilitate the transfer into the full-scale OMT condition, and without any treatment other than a daily supervised dose of buprenorphine-naloxone (24 mg daily on Mondays to Thursdays, with a tripled 72 mg dose on Fridays [31, 32]), that is, without any specific psychosocial treatment. Once a treatment slot in the full-scale OMT program became available, and if the patient could provide a negative urine drug screen, the patient was transferred to the full-scale OMT condition. The obligation to provide a negative urine drug screen was based on interim treatment being considered as an alternative to more traditional treatment entry through in-patient detoxification programs in order to achieve drug abstinence prior to OMT entry. Thus, theoretically, the interim method was considered to potentially facilitate the intake procedure. Drug abstinence during the interim phase was monitored through urine samples taken weekly, unannounced and on varying days. Samples positive for benzodiazepines were handled in the same way in the estimations regardless of whether the substance had been acquired illicitly or through a legal prescription. Only two of the patients who were ever positive for benzodiazepines in urine tests received legal prescriptions for benzodiazepines at some time during the present study.

Patients were not allowed to continue the interim period if they presented extensive illicit use of opioids or other drugs, in case of repeated attempts to manipulate urine tests, or if they were absent for more than seven days, consistent with national regulations for OMT in Sweden. Patients with concomitant drug intake comprising a medical risk were not allowed to enter into full-scale OMT. If patients failed to provide a negative urine sample within three weeks from the offer of an available treatment slot, patients were discharged and advised to apply for OMT from treatment providers outside of the study setting, or to apply for other treatments through social services. In the full-scale OMT program, urine samples were taken regularly one to three times a week. In accordance with Swedish OMT regulations, patients could be involuntarily discharged in case of not showing up for seven consecutive days, or because of a pronounced polydrug use pattern constituting a significant medical risk to the patient.

The outcome measure in the present follow-up study was retention in treatment at follow-up nine months after intake into OMT including the interim phase. Retention implies that the patient voluntarily remains in treatment and is not involuntarily discharged by the program staff. The application of the present discharge criteria follows Swedish regulations for OMT.

As some patients (*n* = 8) participated in the interim treatment on more than one occasion before transition, potential correlates of retention were derived from the last (and successful) attempt in interim treatment. Retention was significantly associated neither with time spent in the interim condition, nor with the variable describing whether the patient entered through their first interim attempt or not (data not shown).

In order to suggest potential correlates of retention for future larger studies, a number of baseline and interim-phase factors were assessed and compared between remaining patients and dropouts. Potential correlates of outcome were the following: age, gender, living conditions (stable housing versus homeless), marital status (married or living together versus living alone), origin (country of birth, Sweden versus other), previous drug overdose, previous suicide attempt, and detailed drug use pattern for the last 30 days prior to baseline and the AUDIT score. An AUDIT score equal to or higher than six for women and eight for men was considered to represent hazardous alcohol use [33]. Variables were run against the outcome measure in univariate analyses.

Continuous variables are presented as median values and interquartile ranges. Comparisons between groups are assessed using the Mann-Whitney* U* test. Categorical variables are presented as absolute and relative frequencies, and comparisons between groups are made using a chi-square test. A *p* value < 0.05 was considered statistically significant. A *p* value > 0.05 but <0.10 was considered to indicate a statistical trend. All *p* values are two-tailed. All calculations were performed using IBM SPSS Statistics for Mac, version 22.

## 3. Results 

Out of 57 patients originally included in the interim study, 40 patients (70%) were successfully transferred through the interim condition to the full-scale OMT condition.

Thirty-six of the patients entering full-scale OMT program had a follow-up time of at least nine months. Out of these 36 patients, 28 patients (78 percent) entered after their first interim period. The median time spent in the interim phase was 35 days. The median age of included subjects was 33 years, and 89 percent were men. The substances most commonly used during the 30 days prior to study inclusion were buprenorphine (75 percent), heroin (72 percent), benzodiazepines (69 percent), cannabis (53 percent), and methadone (53 percent). AUDIT scores were generally low (median 5), whereas 31 percent reached hazardous drinking levels. Previous opioid overdoses and suicide attempts were reported by 69 and 31 percent, respectively.

Thirty patients (83 percent) remained in treatment after nine months. The six dropouts who were discharged during follow-up were all in ongoing illicit substance use and were either actively discharged due to treatment regulations (four cases) or voluntarily discharged (two cases). Retention was furthermore not significantly predicted by age, gender, origin, living conditions, marital status, previous in-patient psychiatric treatment, and previous overdoses or suicide attempts. Nor were there any significant associations between retention and self-reported intake of heroin, methadone, buprenorphine, benzodiazepines, cannabis, amphetamine, or cocaine prior to study start (Table 1).

In the analysis of interim urine samples as potential correlates of nine-month retention, dropout tended to be negatively associated with benzodiazepine use, with a median of 60% positive samples for the dropouts and 27% for retained subjects (*p* = 0.09). There were no significant associations between retention and interim-phase urine samples positive for any of the other drugs (Table 2). In the analysis of urine samples within the full-scale OMT program as correlates of OMT retention, the only type of substance significantly associated with dropout was benzodiazepines, with 4% positive samples for remaining patients and 23% for dropouts (*p* < 0.01, Table 3).

## 4. Discussion 

The present study is, to the best of the authors' knowledge, the first study to report follow-up of patients initiated in OMT through an interim treatment procedure using buprenorphine-naloxone instead of methadone. Although in a small study sample, in patients successfully referred from the interim phase into full-scale treatment, retention through nine months was high. Thus, in this study sample of patients successfully referred from interim medication-only treatment with buprenorphine-naloxone, retention in full-scale OMT was comparable to figures previously reported from the same setting [34].

The use of benzodiazepines during interim treatment and during full-scale OMT was the only variable associated with dropout in the present study. Although the number of dropouts in this pilot study was low, urines positive for benzodiazepines in the interim condition tended to be associated with a negative outcome once referred to the full-scale program but did not reach statistical significance (*p* = 0.09). However, significantly, a negative outcome in full-scale OMT was associated with the use of benzodiazepines within that OMT treatment setting, which was not the case for any other substance, suggesting that benzodiazepines may play a major role in the clinical picture of patients with a negative treatment course in opioid dependence.

The seemingly negative association between retention and benzodiazepine use during treatment of opioid dependence may require further attention in research and in clinical practice. In the present study, at baseline, the frequency of use of benzodiazepines was comparable to that of the main drug of these primarily opioid-dependent subjects. In contrast to the high rates of continued benzodiazepine use, urine samples positive for opiates were very infrequent in the full-scale OMT phase, markedly lower than in many other studies [35, 36], and opioid-positive urines were not associated with dropout in OMT. The role of benzodiazepines in the present results, compared to the role of opioids, strengthens the impression that polydrug use, particularly including the use of benzodiazepines, may present a potentially even larger challenge in the treatment of severe opioid dependence than the actual primary opioid-related disorder.

Patients with a high level of benzodiazepine use could represent a group with more complicated psychiatric problems and more severe substance-related problems [29, 37]. In the present study, patients dropping out of treatment did not report more use of benzodiazepines during the last 30 days prior to study start, but still, this type of substance use was associated with a negative outcome once in full-scale treatment. It cannot be excluded that intake of benzodiazepines actually increases when the intake of illicit opioids decreases in treatment, at least in a subset of individuals.

Previous literature related to polydrug use in OMT patients has shown conflicting findings as to whether the use of nonopioid substances predicts treatment failure or not. Patients with continued use of benzodiazepines [16, 17] or cocaine [24, 25, 27] during OMT have been reported to be more likely to continue using heroin. However, with respect to treatment outcome in OMT, results regarding the risk of polydrug abuse have been ambiguous. While cocaine use also seems to be associated with lower retention in treatment [14, 27, 28], studies on the consequences of benzodiazepine use on treatment outcome have shown conflicting findings, indicating either an association with poor treatment results [29] or no negative effect on treatment outcome [16, 17]. Furthermore, neither cannabis use nor hazardous alcohol use as measured with the AUDIT seems to be associated with treatment outcome [21, 23, 27]. The present study adds to the previous literature on the topic and specifically addresses this issue in the sensitive early treatment course in patients entering OMT through a facilitating model for treatment entry.

In addition to its potential association with dropout in the present pilot study, the high prevalence of benzodiazepine use in the present study is worrying. The couse of benzodiazepines has been reported as a risk situation for opioid overdose among patients in OMT [16]. Also, benzodiazepines are believed to contribute to opioid overdose in a large number (up to 80 percent) of methadone- and buprenorphine-related deaths [16, 17].

Considering the difficulties with excessive and illicit use of benzodiazepines, all positive urine samples in this study were handled as positive, whether or not the patient had a prescription. Thus, without specific information about the reasons for benzodiazepine use in different individuals, its couse within the present treatment context for opioid dependence clearly was associated with an increased risk of treatment failure. It cannot be excluded that treatment retention could be further improved through extended psychosocial support and psychiatric investigations for correct diagnostics. More research is needed in order to further elucidate the mechanisms surrounding the role of benzodiazepine use in treatment dropout seen here. The abuse potential of benzodiazepines has been well-documented, including both the misuse of prescription doses and the actual illicit use of nonprescribed benzodiazepines [38]. While being beyond the scope of the present study, future research should aim to conduct a further in-depth analysis of the differences in abuse potential and consequences of use in this setting for different types of benzodiazepines. In the present setting, flunitrazepam and nitrazepam are among the benzodiazepines most commonly detected in fatal intoxication cases [39], although recent reporting suggests that clonazepam abuse is increasing [40] and that its misuse occurs in substance users, along with alprazolam and diazepam [41].

The present study did not identify any other significant correlates of outcome regarding, for example, age, gender, origin, previous overdoses, or suicide attempts. Our results, although in a small dataset, are consistent with two recent studies, which did not demonstrate correlations between psychiatric severity and compliance in OMT [42, 43]. On the other hand, Coviello and coworkers demonstrated more successful results in retreatment for previously discharged patients among females, nonmarried individuals, and individuals with higher numbers of psychiatric hospitalizations [44]. In one study, younger age and polysubstance abuse at intake were significantly associated with higher risk of involuntary discharge from the program, but AUDIT score was not [45]. All in all, it is hard to find explicit correlates of treatment outcome among baseline characteristics in the literature, and more extensive conclusions are difficult to draw from the present small pilot study. Also, the need for further research applies to the question about whether reentry of patients with initial treatment failures may be associated with treatment outcome. In the present study, reentry (although in very few individuals) was not associated with dropout. This lends support to continued efforts facilitating further treatment attempts in subjects with earlier failure to enter treatment, through an interim approach or other facilitating procedures for treatment initiation. Intuitively, a prompt repeated attempt to include a patient dropping out of treatment is crucial [44].

The present study is limited by the small sample size, and as dropout during nine-month follow-up was low, only six subjects ended up in the dropout group. Given the low sample size, statistical analyses were primarily intended to be of descriptive nature or to suggest potential correlates to assess in larger future trials, that is, partly with a hypothesis-generating approach. For these reasons, only univariate analyses were performed. Clearly, results shall be interpreted with great caution, and the results regarding benzodiazepines can be seen rather as suggesting a role of this type of substance use in the outcome of OMT in opioid dependence, calling for further research in larger samples. Another limitation is that the number of females in the present study was low, compared to other studies assessing interim treatment in opioid dependence [3, 6, 7, 9]. Also, results cannot readily be generalized to patients who are immediately considered to require methadone as maintenance treatment, as the present study was part of an interim treatment project aiming to assess the interim condition with the potentially safer buprenorphine-naloxone medication. Thus, theoretically, the present results may not necessarily apply to all opioid-dependent patients in need of OMT. On the other hand, the clinical severity in the patients enrolled here is likely to be comparable to other treatment studies in patients with opioid dependence, with a high level of complications and with low levels of employment and permanent housing [46–48].

## 5. Conclusion

In a pilot follow-up study of patients successfully referred from interim buprenorphine-naloxone treatment into full-scale OMT, retention throughout a nine-month follow-up period was high. Dropout was associated with the use of benzodiazepines within full-scale treatment, and benzodiazepine use during interim treatment was also suggested (despite not reaching statistical significance) as potentially associated with dropout once in the full-scale maintenance program. Future studies need to further assess the role of benzodiazepines in the treatment of opioid dependence.

## Figures and Tables

**Table 1:** Characteristics of subjects with and without 9-month retention in OMT after transition from buprenorphine interim phase.

	Total	Retention > 9 months	Retention < 9 months	*p*
Number of patients	36	30	6	
Age, median (IQR)	33 (29–42)	32 (29–42)	35 (30–50)	0.419
Male, % (*n*)	89% (32)	90% (27)	83% (5)	0.635
Born in Sweden, % (*n*)	86% (31)	83% (25)	100% (6)	0.281
Previous overdose, % (*n*)	69% (24)	66% (19)	83% (5)	0.392
Previous attempt to commit suicide, % (*n*)	31% (11)	31% (9)	33% (2)	0.912
Days spent in interim phase, median (IQR)	35 (24–50)	35 (25–50)	31 (20–55)	0.756
Substance use in past 30 days, % (*n*)				
Heroin	72% (26)	70% (21)	83% (5)	0.506
Methadone	53% (19)	50% (15)	67% (4)	0.455
Buprenorphine	75% (27)	77% (23)	67% (4)	0.606
Buprenorphine-naloxone	25% (9)	27% (8)	17% (1)	0.606
Benzodiazepines	69% (25)	67% (20)	83% (5)	0.418
Cannabis	53% (19)	53% (16)	50% (3)	0.881
Amphetamine	36% (13)	27% (11)	33% (2)	0.877
Cocaine	6% (2)	7% (2)	0% (0)	0.515
Number of days of substance use in past 30 days, *n* (IQR)				
Heroin	3 (0–13)	3 (0–13)	7 (2–17)	0.519
Methadone	1 (0–4)	1 (0–3)	1 (0–17)	0.514
Buprenorphine	11 (1–27)	11 (1–26)	11 (0–28)	0.915
Buprenorphine-naloxone	0 (0-1)	0 (0-1)	0 (0–3)	0.696
Benzodiazepines	4 (0–14)	4 (0–16)	6 (2–15)	0.517
Cannabis	2 (0–9)	2 (0–8)	1 (0–10)	0.689
Amphetamine	0 (0-1)	0 (0-1)	0 (0–3)	0.901
Cocaine	0 (0-0)	0 (0-0)	—	0.521
AUDIT, hazardous use, % (*n*)	31% (11)	30% (9)	33% (2)	0.871
AUDIT score, median (IQR)	5 (1–10)	5 (1–11)	4 (1–14)	0.881

**Table 2:** Interim urine samples in relation to 9-month retention in OMT.

	Total	Retention > 9 months	Retention < 9 months	*p*
Number of patients	36	30	6	
Percent positive urine samples in IT, median (IQR)				
Opiates	0 (0–23)	0 (0–17)	13 (0–57)	0.393
Benzodiazepines	33 (0–59)	27 (0–56)	60 (19–93)	0.087
Cannabis	6 (0–69)	18 (0–70)	0 (0–37)	0.297
Amphetamine	0 (0–6)	0 (0–9)	0 (0–4)	0.577
Cocaine	0 (0-0)	0 (0-0)	—	0.239

**Table 3:** Urine samples in full-scale OMT in relation to 9-month retention.

	Total	Retention > 9 months	Retention < 9 months	*p*
Number of patients	36	30	6	
Percent positive urine samples, median (IQR)				
Opiates	1 (0–4)	1 (0–4)	2 (0–10)	0.930
Benzodiazepines	6 (2–20)	4 (2–16)	23 (13–43)	0.006
Cannabis	4 (0–20)	4 (0–16)	11 (0–28)	0.965
Amphetamine	1 (0–5)	1 (0–5)	1 (0–13)	0.894
Cocaine	0 (0-1)	0 (0-0)	1 (0–4)	0.105
